# Evaluation of the Synergistic Effect of Tomatidine with Several Antibiotics against Standard and Clinical Isolates of *Staphylococcus aureus*, *Enterococcus faecalis*, *Pseudomonas aeruginosa* and *Escherichia coli*

**Published:** 2017

**Authors:** Rasool Soltani, Hossein Fazeli, Rahim Bahri Najafi, Alireza Jelokhanian

**Affiliations:** a *Department of Clinical Pharmacy and Pharmacy Practice, School of Pharmacy and Pharmaceutical Sciences, Isfahan University of Medical sciences, Isfahan, Iran.*; b *Department of Microbiology, School of Medicine, Isfahan University of Medical sciences, Isfahan, Iran.*; c *Department of Pharmaceutics, School of Pharmacy and Pharmaceutical Sciences, Isfahan University of Medical sciences, Isfahan, Iran. *; d *Department of Clinical Pharmacy and Pharmacy Practice, School of Pharmacy and Pharmaceutical Sciences, Isfahan University of Medical sciences, Isfahan, Iran.*

**Keywords:** Antibiotics, Tomatidine, Synergistic Effect, Bacteria

## Abstract

Antibiotic resistance is an important problem in antibiotic treatment of infections, particularly in hospitals. Tomatidine is a plant secondary metabolite with antimicrobial and antifungal effects. This study examined the possible synergistic effect tomatidine with several antibiotics against standard and clinical strains of *Staphylococcus aureus*, *Enterococcus faecalis*, *Pseudomonas aeruginosa* and *Escherichia coli*. After determining the minimum inhibitory concentrations (MICs) of antibiotics and tomatidine against the bacterial isolates using broth microdilution method, the synergistic effect between tomatidine and antibiotics was evaluated by checkerboard method and calculation of FIC indices. Tomatidine alone did not show any antimicrobial effect. However, it had synergistic effect with gentamicin and cefepime against standard and clinical isolates of *S. aureus* and *P. aeruginosa*, respectively. It also had synergistic effect with ampicillin and ciprofloxacin only against standard strains of *E. faecalis* and *P. aeruginosa*, respectively. In conclusion, tomatidine could be considered as a potential antibiotic potentiator for gentamicin, cefepime and ciprofloxacin, and ampicillin against *Staphylococcus aureus*, *Pseudomonas aeruginosa*, and *Enterococcus faecalis* infections, respectively. However, the toxicological and pharmacological properties of tomatidine for use as a therapeutic agent remain to be determined.

## Introduction

Bacterial infections resistant to antibiotic treatment are one of the major factors threatening the health of human beings. More than 70% of nosocomial pathogens have become resistant to the drugs considered to be their first line treatment (1). Antibiotic resistance is an important issue in the community and particularly in hospitals (2). In the last 50 years, the number of bacterial strains resistant to antibiotics has increased almost uniformly around the world. The bacteria have become resistant to antimicrobial agents by changing their chromosomes and exchanging their genetic materials through plasmids (3). It is clear that the increasing antibiotic resistance can be attributed to the excessive use of these drugs (4).

Gram-positive bacteria, especially Gram-positive cocci such as *Staphylococcus aureus*, enterococci and *Streptococcus pneumoniae*, are the most important pathogens in the hospital environment (5). In the past decade, the percentage of drug-resistant strains of these bacteria has increased (6-7). Increased resistance in Gram-positive bacteria could be a consequence of using complex treatments that also has increased mortality (5).

*Staphylococcus aureus* is Gram-positive facultative aerobic coccus which is the most important species in the *Staphylococcus* genus (8). These bacteria are among the acquired nosocomial pathogens with high prevalence worldwide that are responsible for a wide range of infections including simple skin infections to life-threatening diseases such as pneumonia, meningitis, osteomyelitis and septicemia (9). In a study conducted in 2008 in Imam Khomeini Hospital of Tehran, Iran, *Staphylococcus aureus* strains isolated from clinical specimens of hospitalized patients showed high resistance to most of the evaluated antibiotics (penicillin 95%, oxacillin 60%, cephalexin 42%, cefazolin 53%, co-trimoxazole 61%, ciprofloxacin 42% and gentamicin 45%) (10).

Enterococci are Gram-positive cocci responsible for several nosocomial infections, especially in the intensive care unit (ICU) (11). In the past two decades, enterococci strains resistant to antibiotics such as vancomycin have increased in hospitalized patients (12). 

*Pseudomonas aeruginosa* is among the other bacteria that are commonly present in hospital moist environments and are widely found in nature. *P. aeruginosa* is in the second place among Gram-negative pathogens causing nosocomial infections (13). Currently, most strains of *P. aeruginosa* have intrinsic resistance to many antibiotics such as beta-lactams, tetracyclines, chloramphenicol, and fluoroquinolones (14).

Some herbal compounds exhibit antibiotic-like activity against a wide range of pathogenic bacteria which may put forward a new basis for the development of antimicrobial drugs (15). Nearly a century ago, the antimicrobial activity of plants was studied and used the beneficial results, they were practically used in the treatment of infectious diseases. Nowadays, screening of herbal extracts for the discovery of new drugs is considered by researchers. So far, the antimicrobial properties of many extracts from medicinal plants have been confirmed by laboratory methods (16). These herbal compounds can directly inhibit or destroy the pathogenic bacteria. However, some of them may increase the antimicrobial activity of already known antibiotics (antibiotic potentiator) or change the pathogen’s virulence (virulence attenuator) (15).

The steroidal alkaloids and glycosides are known as compounds with antimicrobial and antifungal activities. There are many plants in the Solanaceae family such as tomato, potato and eggplant that have steroidal alkaloids where the prime example is tomatidine. Tomatidine, the aglycone derivative of tomatine, is a plant secondary metabolite with antimicrobial and antifungal effects (17). Moreover, its synergistic effect with aminoglycosides against drug-resistant strains of *Staphylococcus aureus* has been proven (18). 

Due to the growing resistance of some pathogens to antibiotics, an important factor in failure of treatment of infections, finding new and effective antibiotics or other substances that have the ability to increase the effectiveness of widely used antibiotics is very necessary. The purpose of this study was to investigate the synergistic effect of tomatidine with the most important antibiotics used for infections caused by *Staphylococcus aureus*, enterococci, *Pseudomonas aeruginosa* and *Escherichia coli*. In case the synergistic effect is confirmed, tomatidine can be considered as a reinforcing agent for each of the tested antibiotics against each of the aforementioned pathogens in clinical studies and if it is effective, it will help reducing the dose of antibiotics and further success of treatment.

## Experimental

The study was performed in microbiology laboratory of faculty of Pharmacy and Pharmaceutical Sciences of Isfahan University of Medical Sciences, Isfahan, Iran, from August to December 2014. The evaluated standard strains of microorganisms included *Staphylococcus aureus* (ATCC 29213), *Enterococcus faecalis* (ATCC 29212), *Pseudomonas aeruginosa* (ATCC 27853) and *Escherichia coli* (ATCC 25922). The standard bacterial strains were purchased from the Culture Collection of Industrial and Infectious Microorganisms of Iran. Clinical strains of microorganisms were obtained from isolates cultured from the clinical samples of patients hospitalized in Al-Zahra Hospital of Isfahan, Iran, including wound secretions, sputum, and urine. Tomatidine was purchased from Sigma Corporation of St. Louis, U.S.A. The antibiotic injection preparations from different pharmaceutical companies were used for experiments. They were purchased as follows: cefazolin, ceftazidime, cefepime, and cefuroxime from Loghman, Iran; ampicillin, piperacillin/tazobactam, imipenem, and ceftriaxone from Jaber Ebne Hayyan, Iran; vancomycin from Dana, Iran; Gentamicin from Alborz Darou, Iran; ciprofloxacin from Ronakpharm, Iran; co-trimoxazole from Caspian Tamin, Iran; and teicoplanin from Sanofi Aventis, Switzerland. 

*Broth Microdilution Method for Determination of MIC*

Broth microdilution techniques were performed for determining the minimum inhibitory concentration (MIC) of the antibiotics and tomatidine using CLSI (Clinical Laboratory Standard Institute) guideline (19). The antibiotics used were as follows: *Staphylococcus aureus*: cloxacillin, cefazolin, vancomycin and gentamicin; *Enterococcus faecalis*: teicoplanin, vancomycin, ampicillin and gentamicin; *Pseudomonas aeruginosa*: ceftazidime, tazocin, cefepime, imipenem and ciprofloxacin; *Escherichia coli*: ciprofloxacin, gentamicin, ceftriaxone, cefuroxime and co-trimoxazole. Only the strains sensitive to all related antibiotics were selected for tests.

Each 96-well plate (SPL, South Korea) consists of 8 vertical rows (A to H) and 12 horizontal rows (1 to 12). In each plate, one microorganism was tested. Each vertical row included 5 consecutive 2-fold dilutions of each compound in 2 repeats. The first vertical row was considered as the positive control (containing culture medium and the microbial suspension) and the last vertical row as the negative control (containing culture medium alone). In the first step, 180 µL of Mueller-Hinton broth (Merck, Germany) was added to all wells except for the row 12 which was considered as the negative control. For the row 12, the amount was 200 µL. Then, 20 µL of microbial suspension of 10^6^ CFU/mL was added to each of the wells. The suspension was prepared using the overnight culture of the microorganism and 0.9% sterile saline with the concentration confirmed using a spectrophotometer (at the wavelength of 570 nm and absorption of 0.3). In the next step, the desired concentrations of the tested antibiotics were added. For the preparation of the stock solutions, the powders of cloxacillin, cefazolin, vancomycin, teicoplanin, ampicillin, piperacillin-tazobactam, ciprofloxacin, ceftriaxone, and cefuroxime were dissolved in distilled water while ceftazidime was dissolved in sodium carbonate and cefepime and imipenem were dissolved in phosphate buffer (pH = 7.2). Tomatidine was solubilized with the concentration of 2 g/L in DMSO (Dimthyl sulfoxide) while warmed at 70 °C during the solubilization process. All steps were repeated 3 times for each compound against each microorganism separately and the results were recorded. After incubation at 37 °C for 16-18 h (24 h for vancomycin and teicoplanin), the turbidity was read at wavelength of 540 nm using ELISA Reader (BIO-TEK, U.S.A) (20). The first well in which no turbidity was observed was considered as the minimum inhibitory concentration (MIC). To investigate the synergistic effect in cases where tomatidine did not show any inhibitory effect on the microorganism, the concentration of 32 μg/mL was considered as the minimum inhibitory concentration of this substance (18).

*The antimicrobial effects of antibiotics in combination with tomatidine*

Checkerboard method was used to determine the antimicrobial effects of the tested antibiotics in combination with tomatidine. For this, using 96-well plates, consecutive 2-fold dilutions of each antimicrobial agent were placed in the horizontal row wells and those of tomatidine in the vertical row wells. The inoculated wells containing the culture medium and increasing concentrations of antimicrobial agents from zero to MIC concentration were organized so that all the possible mixtures could be tested. In the first well (lower left corner) as the control, none of the compounds were present while in the last well (upper right corner) there were the highest concentrations of the antimicrobial agent and tomatidine. The first column on the left as well as the first bottom contained only one of the compounds. After filling the wells, the plates were incubated for 16-18 h (24 h for tests of vancomaycin and teicoplanin) at 37 °C. After this period, the ELISA Reader was used to assess growth. To investigate the result of the effect, FIC index (fractional inhibitory concentration) was calculated using the following equation (21):

FIC index = FIC_A_ + FIC_B_ = A / MIC_A_ + B / MIC_B_

Where A is the MIC of the compound A in combination with the compound B, B is the MIC of the compound B in combination with the compound A, MIC_A_ is the minimum inhibitory concentration of the compound A alone and MIC_B_ is the minimum inhibitory concentration of the compound B alone. Considering the FIC index, the effect of the two compounds on each other can be determined, so that if the index is ≤0.5, the two substances have synergistic effect, if >0.5 and ≤4, the two substances do not affect each other and if >4, the two substances have antagonistic effect on each other (22).

## Results and Discussion

Tomatidine alone showed no inhibitory effect on the tested standard and clinical isolates up to a concentration of 200μg/mL. Table 1. shows the minimum inhibitory concentrations of evaluated antibiotics as well as the results of the synergy tests of each one with tomatidine against the standard bacterial strains. As shown, tomatidine had synergistic effect with gentamicin against *Staphylococcus aureus*, with ampicillin against *Enterococcus faecalis* and with cefepime and ciprofloxacin against *Pseudomonas aeruginosa*, while it showed no synergistic effect with any of the tested antibiotics against *Escherichia coli*.

Table 2. shows the minimum inhibitory concentrations of evaluated antibiotics as well as the results of the synergy tests of each one with tomatidine against the clinical bacterial strains. As shown, tomatidine had synergistic effect with gentamicin against *Staphylococcus aureus* and with cefepime against *Pseudomonas aeruginosa*, while it showed no synergistic effect with any of the tested antibiotics against *Enterococcus faecalis* and *Escherichia coli*.

Various studies have been conducted on herbal compounds as antibiotic potentiators, which increased the sensitivity of bacteria to antibiotics. For example, reports have shown that thymol and carvacrol have synergistic effects with ampicillin, tetracycline, penicillin, bacitracin and erythromycin (23).

**Table 1:** The MIC values of tested antibiotics against standard strains of microorganism and the results of synergy test for combination of each antibiotic with tomatidine

**Microorganism**	**antibiotics**	**MIC ** **(** **μg/mL)**	**FIC**	**Synergistic effect with tomatidine**
*S. aureus*	Cloxacillin	0.12	>0.5	No
	Cefazolin	0.25	>0.5	No
	Vancomycin	0.5	>0.5	No
	Gentamicin	1	0.37	Yes
*E. faecalis*	Teicoplanin	0.25	>0.5	No
	Vancomycin	1	>0.5	No
	Ampicillin	0.5	0.49	Yes
	Gentamicin	8	>0.5	No
	Ceftazidime	2	>0.5	No
	Piperacillin-tazobactam	1	>0.5	No
*P. aeruginosa*	Cefepime	4	0.5	Yes
	Imipenem	1	>0.5	No
	Ciprofloxacin	0.06	0.5	Yes
*E. coli*	Ciprofloxacin	0.015	>0.5	No
	Gentamicin	1	>0.5	No
	Ceftriaxone	0.03	>0.5	No
	Cefuroxime	2	>0.5	No
	Trimethoprim-sulfamethoxazole	0.5	>0.5	No

**Table 2:** The MIC values of tested antibiotics against clinical strains of microorganism and the results of synergy test for combination of each antibiotic with tomatidine

Microorganism	antibiotics	MIC (μg/mL)	FIC	Synergistic effect with tomatidine
*S. aureus*	Cloxacillin	0.25	>0.5	No
	Cefazolin	0.5	>0.5	No
	Vancomycin	0.5	>0.5	No
	Gentamicin	2	0.375	Yes
*E. faecalis*	Teicoplanin	1	>0.5	No
	Vancomycin	2	>0.5	No
	Ampicillin	8	>0.5	No
	Gentamicin	16	>0.5	No
	Ceftazidime	8	>0.5	No
	Piperacillin-tazobactam	4	>0.5	No
*P. aeruginosa*	Cefepime	8	0.5	Yes
	Imipenem	2	>0.5	No
	Ciprofloxacin	0.12	>0.5	No
*E. coli*	Ciprofloxacin	0.03	>0.5	No
	Gentamicin	1	>0.5	No
	Ceftriaxone	0.12	>0.5	No
	Cefuroxime	8	>0.5	No
	Trimethoprim-sulfamethoxazole	1	>0.5	No

This study showed that tomatidine has synergistic effect with gentamicin against both standard and clinical strains of *Staphylococcus aureus*. It also showed synergistic effect with ampicillin against *Enterococcus faecalis* as well as ciprofloxacin and cefepime against *Pseudomonas aeruginosa* only on the standard strains and did not affect the clinical strains.The synergistic effect was also observed with cefepime against both standard and clinical isolates of *P. aeruginosa.* In fact, our study is the first work evaluating the synergism between tomatidine and antibiotics against major pathogens. At the best of our knowledge, there are only two studies about the antimicrobial effects of tomatidine, both conducted by Mitchell *et al*. The first study showed that tomatidine has growth inhibitory activity against small-colony variants (SCVs) of *S. aureus* with MIC of 0.12 μg/mL while the growth of normal strains was not affected by tomatidine up to a concentration of 128 μg/mL (24). Although we evaluated the antimicrobial effects of tomatidine up to a concentration of 200 μg/mL, similar to this study, no inhibitory effect was detected against *S. aureus* strains. The second study evaluated the possible synergistic effect of tomatidine with various antibiotics against *S. aureus* strains as well as the aminoglycosides against Enterococci, *P. aeruginosa*, and *E. coli* (18). Consistent to our results, this study showed the potentiating effect of tomatidine on aminoglycosides against both standard and clinical strains of *S. aureus*. However, unlike the mentioned work, we did not evaluated multidrug-resistant strains of *S. aureus*. Based on these results, tomatidine could be considered as a potential antimicrobial agent for use in combination with lower doses of aminoglycosides against various staphylococcal infections; however, *in-vivo* studies are required to confirm this. 

Our results showed potentiating effect of tomatidine for ampicillin against standard strains of *E. faecalis* but not clinical isolates. This could be due to higher resistance of clinical isolates because of their exposure to antibiotics over time. Similar results were observed for ciprofloxacin against *P. aeruginosa*. Considering the observed effects on standard strains, it is possible that higher concentrations of tomatidine could affect these antibiotics against clinical isolates. 

The mechanism of antibacterial and antibiotic potentiating effects of tomatidine is unknown; however, several mechanisms have been proposed for its anti-staphylococcal effect including the inhibition of the biosynthesis of macromolecules especially proteins (24) as well as the inhibition of the expression of virulence factors and biofilm formation (24, 25). Several other studies have shown synergistic effect between natural products and antibiotics against *S. aureus* strains including MRSA isolates with the mechanisms of action being determined such as the synergism between epicallocatechin-gallate and ampicillin/sulbactam through inhibition of bacterial β-lactamase (26), between diterpenes and tetracycline by blockade of MDR (multidrug resistance) pump (27), and between pomegranate extract and chloramphenicol, gentamicin, ampicillin, and oxacillin through inhibition of Nor (A) pump (an efflux pump) (28). 

## Conclusion

Tomatidine could be considered as a potential antibiotic potentiator for gentamicin, cefepime and ciprofloxacin, and ampicillin against *Staphylococcus aureus*, *Pseudomonas aeruginosa*, and *Enterococcus faecalis* infections, respectively. However, the toxicological and pharmacological properties of tomatidine for use as a therapeutic agent remain to be determined. 
